# Spatiotemporal dynamics in excitable homogeneous random networks composed of periodically self-sustained oscillation

**DOI:** 10.1038/s41598-017-12333-3

**Published:** 2017-09-19

**Authors:** Yu Qian, Fei Liu, Keli Yang, Ge Zhang, Chenggui Yao, Jun Ma

**Affiliations:** 1 Nonlinear Research Institute, Baoji University of Arts and Sciences, Baoji, 721007 China; 2 Department of Physics, Lanzhou University of Technology, Lanzhou, 730050 China; 3 Department of Mathematics, Shaoxing University, Shaoxing, 312000 China; 4 King Abdulaziz Univ, Fac Sci, Dept Math, NAAM Res Grp, Jeddah, 21589 Saudi Arabia

## Abstract

The collective behaviors of networks are often dependent on the network connections and bifurcation parameters, also the local kinetics plays an important role in contributing the consensus of coupled oscillators. In this paper, we systematically investigate the influence of network structures and system parameters on the spatiotemporal dynamics in excitable homogeneous random networks (EHRNs) composed of periodically self-sustained oscillation (PSO). By using the dominant phase-advanced driving (DPAD) method, the one-dimensional (1D) Winfree loop is exposed as the oscillation source supporting the PSO, and the accurate wave propagation pathways from the oscillation source to the whole network are uncovered. Then, an order parameter is introduced to quantitatively study the influence of network structures and system parameters on the spatiotemporal dynamics of PSO in EHRNs. Distinct results induced by the network structures and the system parameters are observed. Importantly, the corresponding mechanisms are revealed. PSO influenced by the network structures are induced not only by the change of average path length (APL) of network, but also by the invasion of 1D Winfree loop from the outside linking nodes. Moreover, PSO influenced by the system parameters are determined by the excitation threshold and the minimum 1D Winfree loop. Finally, we confirmed that the excitation threshold and the minimum 1D Winfree loop determined PSO will degenerate as the system size is expanded.

## Introduction

Neuron is the basic unit in neuronal networks and brain systems. The dynamical behaviors of electrical activities in neurons are much complex and have been extensively investigated in the past decades. With the change of intrinsic parameters or external environment, single neuron can present multiple modes of electrical activities, such as quiescent, spiking, bursting, even chaotic states, readers can find possible guidance from the review in Refs ^5,6^. Moreover, the neuronal networks and brain systems can exhibit persistent electrical oscillations at different levels^7–16^. For example, Bazhenov *et al*. studied the self-sustained rhythmic activity in the thalamic reticular nucleus mediated by depolarizing GABA_A_ receptor potentials^7^. Buzsáki *et al*. reviewed the neuronal oscillations in cortical networks^9^. Bartos *et al*. summarized the synaptic mechanisms of synchronized gamma oscillations in inhibitory interneuron networks^10^. Guerriera *et al*. revealed the robust network oscillations during mammalian respiratory rhythm generation driven by synaptic dynamics^15^. Recent experimental studies have shown that these rhythmic activities are related to some specific and important physiological functions *in vivo* ^17–22^. For example, Palva *et al*. found that distinct gamma-band can evoke responses to speech and non-speech sounds in humans^17^. Ward *et al*. summarized the correlation between synchronous neural oscillations and cognitive processes^18^. Bollimunta *et al*. revealed the neuronal mechanisms of cortical alpha oscillations in awake-behaving macaques^19^. Kay *et al*. discovered a beta oscillation network in the rat olfactory system during a 2-alternative choice odor discrimination task^20^. Burke *et al*. reported the synchronous and asynchronous theta and gamma activities during episodic memory formation^21^. Jensen *et al*. disclosed that temporal coding organized by coupled alpha and gamma oscillations can prioritize visual processing^22^.

Theoretically, several famous neuron models, such as the Hodgkin-Huxley neuron model^23^, the Morris-Lecar neuron model^24^, the Hindmarch-Rose neuron model^25^, and the Chay neuron model^26^, have been set up to simulate the neuronal dynamics. Researchers carefully regulate the bifurcation parameters to reproduce the multiple modes of electrical activities in single neuron. Some researchers even suggest that the present neuron models should be improved to include more system parameters and the effect of modulation from astrocyte should also be considered. For example, Tang *et al*. constructed a minimal neuron-astrocyte network model by connecting a neurons chain and an astrocytes chain, and the role of astrocyte on seizure-like discharges was discussed^27^. Lv *et al*. argued that magnetic flux can be used to model the effect of electromagnetic induction and radiation in neurons, and multiple modes in electrical activities were observed^28,29^. Based on these biological and physical neuron models, different types of network connection are considered, thus the transition of collective behaviors in network can be understood. Within this topic, pattern selection and control, synchronization stability are appreciated and are paid much attention^30,31^. In the chain or ring network, wave propagation is often used to discuss the collective behaviors supported by different types of oscillators. Furthermore, the models of excitable complex network are established to investigate the phenomena observed in neuronal networks and brain systems. Self-sustained oscillation is one of the most important issues under investigation in this field due to its extensive application in these systems^32–46^. In recent years, diverse self-sustained oscillatory activities are revealed in different kinds of excitable complex networks. For example, Sinha *et al*. discovered the emergence of self-sustained patterns in small-world excitable media^35^. McGraw *et al*. reported the self-sustaining oscillations in homogeneous random networks of excitable elements^41^. Mi *et al*. studied the long-period rhythmic synchronous firing in a scale-free network containing excitable neurons^42^. The author of present paper discussed the emergence of self-sustained oscillations in excitable Erdös-Rényi random networks^43^.

It has been confirmed that the interactions between neurons in neuronal networks and brain systems are particularly complex, and can constitute complicated structural networks. More importantly, these anatomical complex structures can really influence the dynamic characteristic of coherent physiological activities. Previous researches have shown that there is an important relationship between the network structure and the spatiotemporal dynamics in these two systems^47–55^. For example, Bogaard *et al*. investigated the interaction of cellular and network mechanisms in spatiotemporal pattern formation in neuronal networks^48^. Mäki-Marttunen *et al*. studied the effects of local structure of neuronal networks on spiking activity in silico^49^. Butz *et al*. found that homeostatic structural plasticity can increase the efficiency of small-world neuronal networks^53^. Gonzalez *et al*. discussed the impact of connections between oscillatory neuronal networks on oscillation frequency and pattern^54^. Jovanović *et al*. revealed the interplay between graph topology and correlations of third order in spiking neuronal networks^55^.

In this paper, we systematically investigate the influence of network structures and system parameters on the spatiotemporal dynamics in excitable homogeneous random networks (EHRNs) composed of periodically self-sustained oscillation (PSO). Distinct impacts induced by the network structures and the system parameters are observed. Importantly, the corresponding mechanisms are revealed.

## The Mathematical Model and The Order parameter

In the present paper, we consider the EHRN containing *N* nodes. The Bär-Eiswirth model^56^ is adopted to describe the local kinetics. The evolution of the studied network dynamics is described by the following equations:

1 $$\frac{{\rm{d}}{u}_{i}}{{\rm{d}}t}=-\frac{1}{\varepsilon }{u}_{i}({u}_{i}-\mathrm{1)}({u}_{i}-\frac{{v}_{i}+b}{a})+D\sum _{j\mathrm{=1}}^{N}{A}_{i,j}({u}_{j}-{u}_{i}),$$

2 $$\frac{{\rm{d}}{v}_{i}}{{\rm{d}}t}=f({u}_{i})-{v}_{i},$$

where *i* = 1, 2, …, *N* represents the position of excitable node in the network. In equations (1) and (2), the variables *u*_*i*_ and *v*_*i*_ describe the activator and the inhibitor of the *i*th node, respectively, and the function *f*(*u*) satisfies the form: *f*(*u*) = 0 for $u < \frac{1}{3}$; $f(u)=1-6.75u{(u-\mathrm{1)}}^{2}$ for $\frac{1}{3}\le u\le 1$; and *f*(*u*) = 1 for *u* > 1. Here *ε* is the small relaxation parameter, which represents the time ratio between the activator *u* and the inhibitor *v*. The dimensionless parameters *a* and *b* denote the activator kinetics of the local dynamics and can effectively control the excitation threshold (the excitation threshold of Bär-Eiswirth model is determined by ${u}_{{\rm{th}}}=\frac{b}{a}$). *D* is the coupling strength of activator *u*, which determines the interaction intensity between linking nodes. In Eq. (1), *A*_*i,j*_ is the adjacency matrix element, and is defined as *A*_*i,j*_ = *A*_*j,i*_ = 1 if there is a bidirectional connection linking nodes *i* and *j* in the network, and *A*_*i,j*_ = *A*_*j,i*_ = 0 otherwise. As EHRN is considered in this paper, we adopt identical degree *k* for each node (i.e., each node in the network couples to *k* other nodes, and the bidirectional symmetric couplings are chosen randomly). Here, we should mention that although the Bär-Eiswirth model is originated from chemical systems, it can also exhibit a typically excitable dynamics for appropriate sets of system parameters. Importantly, the parameters used in the present paper can ensure the excitable dynamics of Bär-Eiswirth model. Consequently, the Bär-Eiswirth model can be used to describe typical excitability of neurons, and the network dynamics of equations (1) and (2) can serve as a simplified version of neuronal networks^36,37,42,43,57–59^. In this case variable *u* represents the membrane potential and variable *v* is the somatic inhibitory current. The diffusive coupling simulates electrical conjunction interaction between neurons. The above studied network dynamics are integrated by the forward Euler integration scheme with time step Δ*t* = 0.02. The random initial condition is used in the numerical simulation (i.e., the initial variables ${u}_{i}(t\,=\,\mathrm{0)}$ and ${v}_{i}(t\,=\,\mathrm{0)}$ are randomly given between 0 and 1).

**Figure 1:**
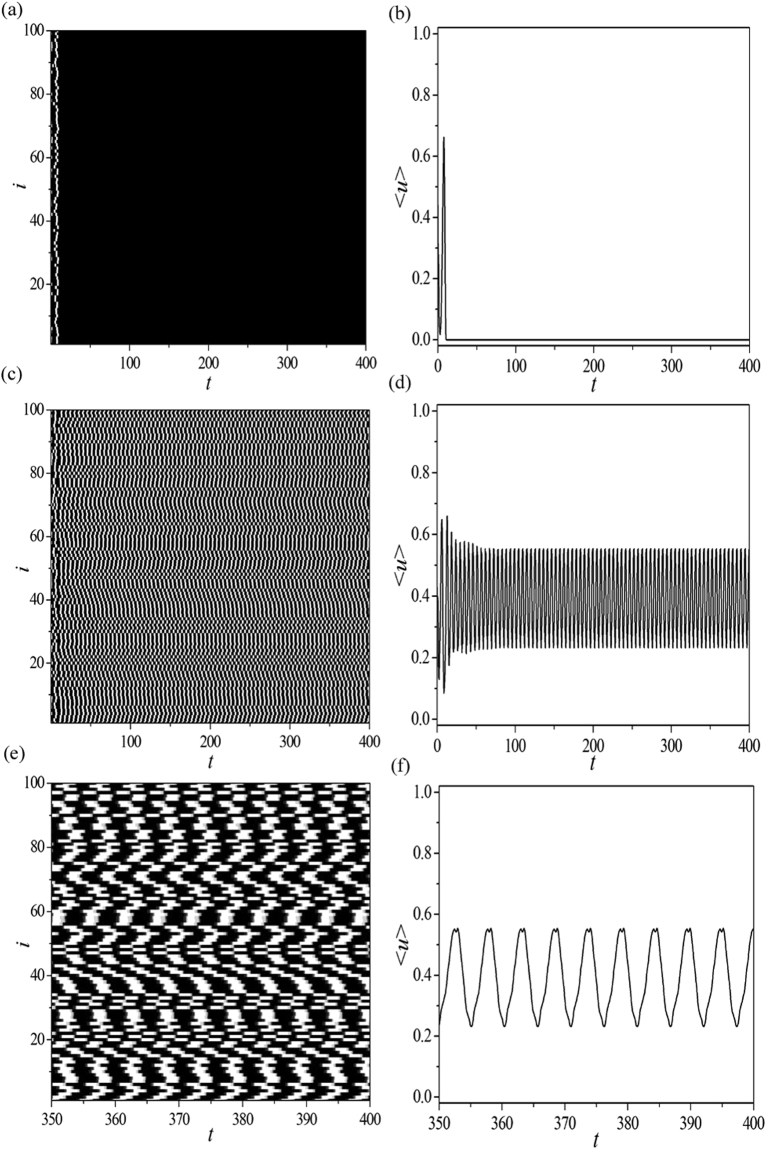
The numerical computation results of the studied excitable homogeneous random networks (EHRNs) for the parameters *a* = 0.90, *b* = 0.04, *ε* = 0.04, *D* = 0.30, *N* = 100 and *k* = 3. Time step Δ*t* = 0.02. (**a**) Spatiotemporal evolution pattern of the rest state realized from a certain set of random initial conditions in EHRNs. The figure is plotted in grayscale from black (lowest value at 0.0) to white (highest value at 1.0). This grayscale will be used throughout this paper. Time passes from left to right. (**b**) The asymptotic time series $< u(t) > =\frac{1}{N}{\sum }_{i\mathrm{=1}}^{N\mathrm{=100}}{u}_{i}(t)$ of pattern (**a**). (**c**) Spatiotemporal evolution pattern of the self-sustained oscillation realized from another set of random initial conditions in EHRNs. (**d**) The asymptotic time series $< u(t) > =\frac{1}{N}{\sum }_{i\mathrm{=1}}^{N\mathrm{=100}}{u}_{i}(t)$ of pattern (**c**). (**e**) (**f**) The local amplifications of panels (c) and (d), respectively. Periodically oscillatory behavior is indicated obviously. This implies that periodically self-sustained oscillation (PSO) can emerge in EHRNs with suitable initial conditions.

In order to quantitatively investigate the influence of network structures and system parameters on the spatiotemporal dynamics of PSO in EHRNs, and also reveal the corresponding mechanisms, the oscillation proportion *p*_os_ is introduced as the order parameter. It is defined as follows:

3 $${p}_{{\rm{os}}}=\frac{{N}_{{\rm{os}}}}{{N}_{{\rm{ALL}}}},$$

where *N*_ALL_ is the total number of numerical simulations performed for each set of parameters and *N*_os_ is the number of PSOs counted in the *N*_ALL_ independent samples. For each simulation, we execute 20000 time steps (i.e., the duration of each simulation is 400 time units), and we utilize the last 200 time units of simulation to judge whether PSO emerges in the EHRN or not. The detailed numerical criterion is as follows. If one of the nodes in the network executes permanently periodical cycles in the last 200 time units (such as the spatiotemporal evolution pattern shown in Fig. 1(c)), PSO is deemed to emerge in EHRN in this numerical simulation. We count this as 1 PSO (i.e., *N*_os_ = 1). Here we should mention that if the EHRN performs successive 37 oscillatory cycles in the last 200 time units in 1 numerical simulation (such as the asymptotic time series shown in Fig. 1(d)), it is still counted as 1 PSO. If nodes in the network are all in the rest state in the last 200 time units (such as the spatiotemporal evolution pattern shown in Fig. 1(a)), no PSO can be observed in this numerical simulation (i.e., *N*_os_ = 0). For each set of parameters, one hundred independent numerical simulations are performed (i.e., *N*_ALL_ = 100). The above criterion is employed to count the number of PSOs *N*_os_ observed in this *N*_ALL_ = 100 independent samples. In the following, we will use oscillation proportion ${p}_{{\rm{os}}}=\frac{{N}_{{\rm{os}}}}{{N}_{{\rm{ALL}}}}$ as the order parameter to investigate the influence of network structures and system parameters on the spatiotemporal dynamics of PSO in EHRNs, and to reveal the corresponding mechanisms.

## Numerical Results and Discussions

### The PSO In EHRN And The Corresponding Oscillation Source

In this part we first study the spatiotemporal dynamics obtained in EHRNs. The numerical computation results of the studied EHRN for parameters *a* = 0.90, *b* = 0.04, *ε* = 0.04, *D* = 0.30, *N* = 100 and *k* = 3 is exhibited in Fig. 1. Figure 1(a) shows the spatiotemporal evolution pattern of the rest state realized from a certain set of random initial conditions. In the white regions, the nodes fire, while in the black ones they are quiescent, and time passes from left to right. The EHRN damps to the homogeneous rest state from the random initial excitations. Figure 1(b) displays the asymptotic time series $< u(t) > =\frac{1}{N}{\sum }_{i\mathrm{=1}}^{N\mathrm{=100}}{u}_{i}(t)$ of pattern Fig. 1(a). It is shown that $< u(t) >$ damps to zero after a spiking. Figure 1(c) shows a distinct spatiotemporal evolution pattern realized from another set of random initial conditions. Nodes in the network exhibit successively excitations. Figure 1(d) exposes the corresponding asymptotic time series $< u(t) > =\frac{1}{N}{\sum }_{i\mathrm{=1}}^{N\mathrm{=100}}{u}_{i}(t)$. Permanently oscillatory behavior of $< u(t) >$ is detected. The local amplifications of Fig. 1(c) and (d) are displayed in Fig. 1(e) and (f), respectively. It is shown obviously that the permanently oscillatory behavior observed in Fig. 1(c) and (d) is periodical, which means that the spatiotemporal dynamics of PSO can emerge in EHRNs with suitable initial conditions.

Now we would ask where is the oscillation source of PSO, and how excitable wave propagates from the oscillation source to the whole network. To answer the above two questions, the dominant phase-advanced driving (DPAD) method^36^, which was proposed to analyze the oscillation source and wave propagation path of oscillatory complex networks consisting of non-oscillatory nodes, will be employed. Here we briefly interpret the main idea of the DPAD method. Given a network consisting of *N* nodes with non-oscillatory local dynamics, there are *M*(*M* > *N*) interactions between different nodes. It is evident that any individual non-oscillatory node can oscillate if and only if it is driven by one or more interactions with advanced phases. Among all phase-advanced interactions, the interaction providing the most contribution to exciting the given node, is defined as the dominant phase-advanced driving. Based on this idea, the corresponding DPAD path for each node can be identified. Then the original oscillatory complex network can be reduced to structurally simple and instructive subnetwork of the DPAD pattern, which can effectively reveal the mechanism of the oscillation.

**Figure 2:**
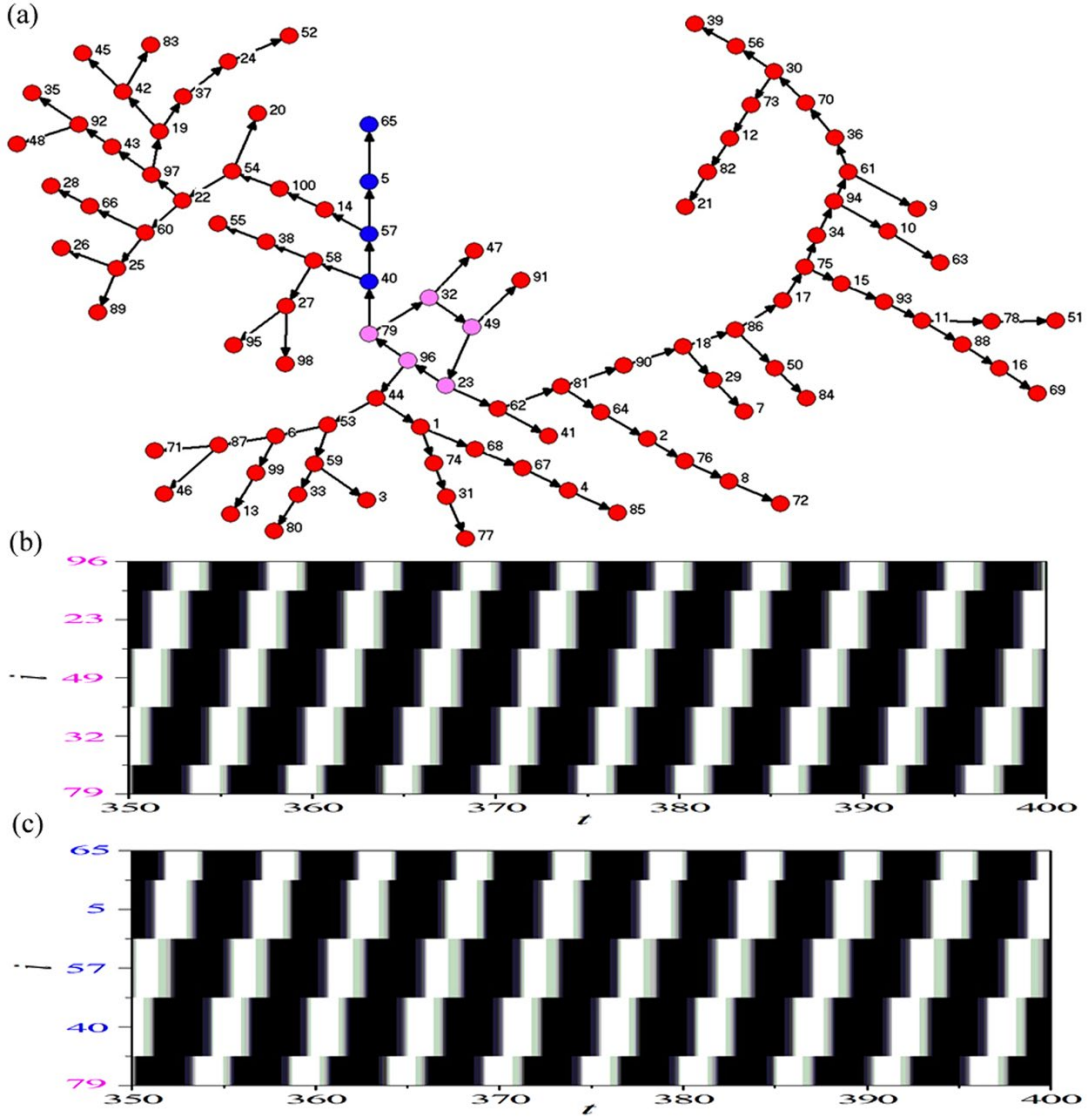
(**a**) The dominant phase-advanced driving (DPAD) pattern corresponding to the PSO of Fig. 1(c). All subscripts indicate the node positions in the network. From the DPAD pattern we can identify the following information: (i) The oscillation source (indicated by the loop structure composed by five pink nodes); (ii) The excitable wave propagation pathways in the network (indicated by the successive arrowed driving sequences). (**b**) Spatiotemporal evolution pattern of nodes in the loop structure. These nodes are ordered according to the sequence in the loop. The one-dimensional (1D) Winfree loop which plays the role of the oscillation source is revealed explicitly. (**c**) Spatiotemporal evolution pattern of nodes in a branch in the DPAD pattern (indicated by blue nodes in panel (a)). The accurate excitable wave propagation pathways from the oscillation source to the nodes in network are exposed clearly.

Figure 2(a) shows the DPAD pattern corresponding to the PSO of Fig. 1(c). Based on the information revealed by the DPAD pattern, the above two questions can be explained without any ambiguity. First, we have exposed in Fig. 2(a) the oscillation source (indicated by the loop structure composed by five pink nodes). Other nodes are positioned on the branches radiated from the loop structure. Figure 2(b) displays the spatiotemporal evolution pattern of nodes in the loop structure. These nodes are ordered according to the sequence in the loop. Successive excitable wave propagation is formed in the loop. This is exactly the self-sustained excitable one-dimensional (1D) Winfree loop^60^, which plays the role of oscillation source supporting the spatiotemporal dynamics of PSO in EHRN. Second, the excitable wave propagation pathways are uncovered (indicated by the successive arrowed driving sequences in Fig. 2(a)). To further prove this point, the spatiotemporal evolution pattern of nodes in a branch in the DPAD pattern (indicated by blue nodes in Fig. 2(a)) is plotted in Fig. 2(c). The accurate excitable wave propagation pathways from the oscillation source to the nodes in network are exposed clearly.

Based on the results revealed in this part we can declare that the spatiotemporal dynamics of PSO can emerge in EHRNs with suitable initial conditions. Importantly, by using the DPAD method, the 1D Winfree loop is revealed as the oscillation source supporting the PSO, and the accurate wave propagation pathways from the oscillation source to the whole network are exposed clearly, which are otherwise deeply hidden in the complicated spatiotemporal evolution pattern.

### The influence of network structures on PSO in EHRNs and the corresponding mechanisms

**Figure 3:**
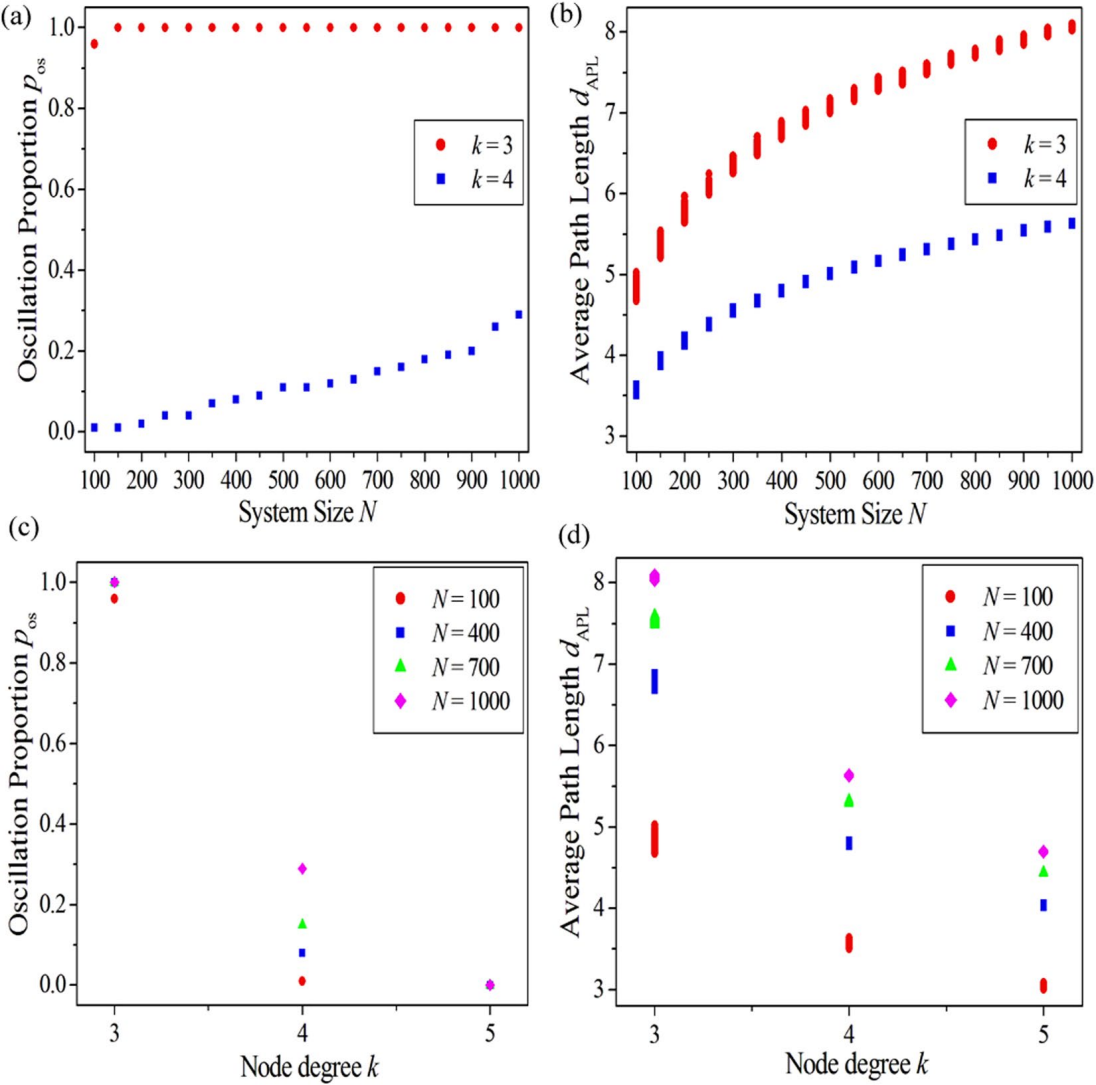
(**a,b**) The dependence of the oscillation proportion ${p}_{{\rm{os}}}$ (**a**) and the average path length (APL) of network ${d}_{{\rm{APL}}}$ (**b**) on the system size $N$ for different node degrees $k$ in EHRNs (shown by red dots for $k\,=\,3$ and shown by blue squares for $k\,=\,4$). (**c,d**) The dependence of the oscillation proportion ${p}_{{\rm{os}}}$ (**c**) and the APL of network ${d}_{{\rm{APL}}}$ (**d**) on the node degree $k$ for different system sizes $N$ in EHRNs (shown by red dots for $N\,=\,100$, shown by blue squares for $N\,=\,400$, shown by green triangles for $N\,=\,700$ and shown by pink diamonds for $N\,=\,1000$). System parameters are chosen as: $a\,=\,0.90$, $b\,=\,0.04$, $\varepsilon \,=\,0.04$ and $D\,=\,0.30$. The oscillation proportion is defined as ${p}_{{\rm{os}}}=\frac{{N}_{{\rm{os}}}}{{N}_{{\rm{ALL}}}}$. Here ${N}_{{\rm{ALL}}}$ is the total number of numerical simulations executed for each set of parameters, and ${N}_{{\rm{os}}}$ is the number of PSOs counted in the ${N}_{{\rm{ALL}}}$ independent numerical simulations. One hundred independent numerical simulations (i.e., ${N}_{{\rm{ALL}}}\mathrm{=100}$) are performed for each set of parameters.

Now we investigate the influence of network structures on the spatiotemporal dynamics of PSO in EHRNs. We mainly focus on the system size *N* and the node degree *k*, which are two major manners in regulating the network structure. Other system parameters are chosen as *a* = 0.90, *b* = 0.04, *ε* = 0.04 and *D* = 0.30 in this part. The oscillation proportion pos is used as the indicator. Figure 3(a) displays the dependence of the oscillation proportion *p*_os_ on the system size *N* for different node degrees *k* in EHRNs. For *k* = 3 (shown by red dots), *p*_os_ increases straightly from 0.96 (corresponding to *N* = 100) to 1.00 (corresponding to $N\ge 150$). For $k=4$ (shown by blue squares), *p*_os_ increases gradually from 0.01 (corresponding to *N* = 100) to 0.29 (corresponding to *N* = 1000). It is shown from Fig. 3(a) that the oscillation proportion *p*_os_ can increase as the system size *N* is increased both for node degrees *k* = 3 and *k* = 4. This means that larger system size is beneficial for the spatiotemporal dynamics of PSO and can promote the emergence of PSOs in EHRNs. The larger the system size is, the more the PSO can emerge in EHRNs.

It is well known that system size can effectively influence the property of network structure, especially in impacting on the average path length (APL) of network. Therefore, the relationship between APL of network and system size is the crucial point to explain the results obtained in Fig. 3(a). Figure 3(b) shows the dependence of the APL of EHRN *d*_APL_ on the system size *N* for two different node degrees *k* (as shown by red dots for *k* = 3 and blue squares for *k* = 4). It is exhibited that the APL of EHRN increases gradually as the system size is increased both for *k* = 3 and *k* = 4. As we know APL denotes the average shortest path between any two nodes in complex network. It can be approximately considered as the distances between the initially excited nodes to their corresponding driving nodes along the wave propagation pathways in the network. When APL is long, the initially excited nodes have enough time to response to the next excitation from their driving nodes, the 1D Winfree loop has a great chance to form, and the PSO can largely emerge in the EHRN. However, the initially excited nodes are largely in the refractory periods as APL is short. The 1D Winfree loop has a little chance to form, or even cannot form in this case. Consequently, the PSO can hardly emerge in the EHRN. Based on the results shown in Fig. 3(a) and (b) and the above analysis we can assert that the system size, which is one of the major manners in regulating the network structure, can effectively influence the spatiotemporal dynamics of PSO in EHRNs. The corresponding mechanism is the change of the APL of network.

Here we discuss the effect of node degree on the spatiotemporal dynamics of PSO in EHRNs. Figure 3(c) exhibits the dependence of the oscillation proportion pos on the node degree k for different system sizes N in EHRNs (as shown by red dots for N = 100, blue squares for N = 400, green triangles for N = 700 and pink diamonds for N = 1000). Contrary to the impact induced by the system size (comparing to the results shown in Fig. 3(a)), the oscillation proportion *p*_os_ decreases remarkably as the node degree *k* is increased. When the node degree *k* is small (i.e., *k* = 3), the oscillation proportions are all in a higher level (i.e., the *p*_os_ almost equals to 1.0 in each system size *N*). This means that the PSO can almost emerge in EHRNs in each numerical simulation for small node degree *k*, which is nearly regardless of the system size. As node degree *k* is increased (i.e., *k* = 4), each oscillation proportion *p*_os_ decreases to a lower level abruptly. Now the system size becomes significantly. It is displayed that the larger the system size is, the more the PSO can emerge in EHRNs. When node degree *k* is further increased (i.e., *k* = 5), oscillation proportions *p*_os_ obtained for different system sizes *N* are all located at zero (i.e., *p*_os_ = 0.0 for all *N*). This indicates that large node degree is harmful for the spatiotemporal dynamics of PSO and can hinder the formation of PSO in EHRNs. No PSO can be observed in this case.

Similar to the system size, node degree can also regulate the network structure by changing the APL of network. Consequently, we can speculate the decrease of the oscillation proportion *p*_os_ induced by the increase of node degree *k* in EHRNs may be caused by the decrease of APL of network. To verify our conjecture, the dependence of the APL of EHRN *d*_APL_ on the node degree *k* for different system sizes *N* is plotted in Fig. 3(d) (as shown by red dots for *N* = 100, blue squares for *N* = 400, green triangles for *N* = 700 and pink diamonds for *N* = 1000). It is displayed that the APL of EHRN *d*_APL_ decreases remarkably as the node degree *k* is increased. As stated above the initially excited nodes are largely in the refractory periods when the APL of network is short. The 1D Winfree loop can hardly form in this case. As a result, fewer PSOs or even no PSO can emerge in EHRNs for large node degree *k*, and the results shown in Fig. 3(c) can be obtained. Now, our conjecture has been confirmed, and a possible mechanism behind the spatiotemporal dynamics of PSO in EHRNs influenced by the node degree is revealed.

**Figure 4:**
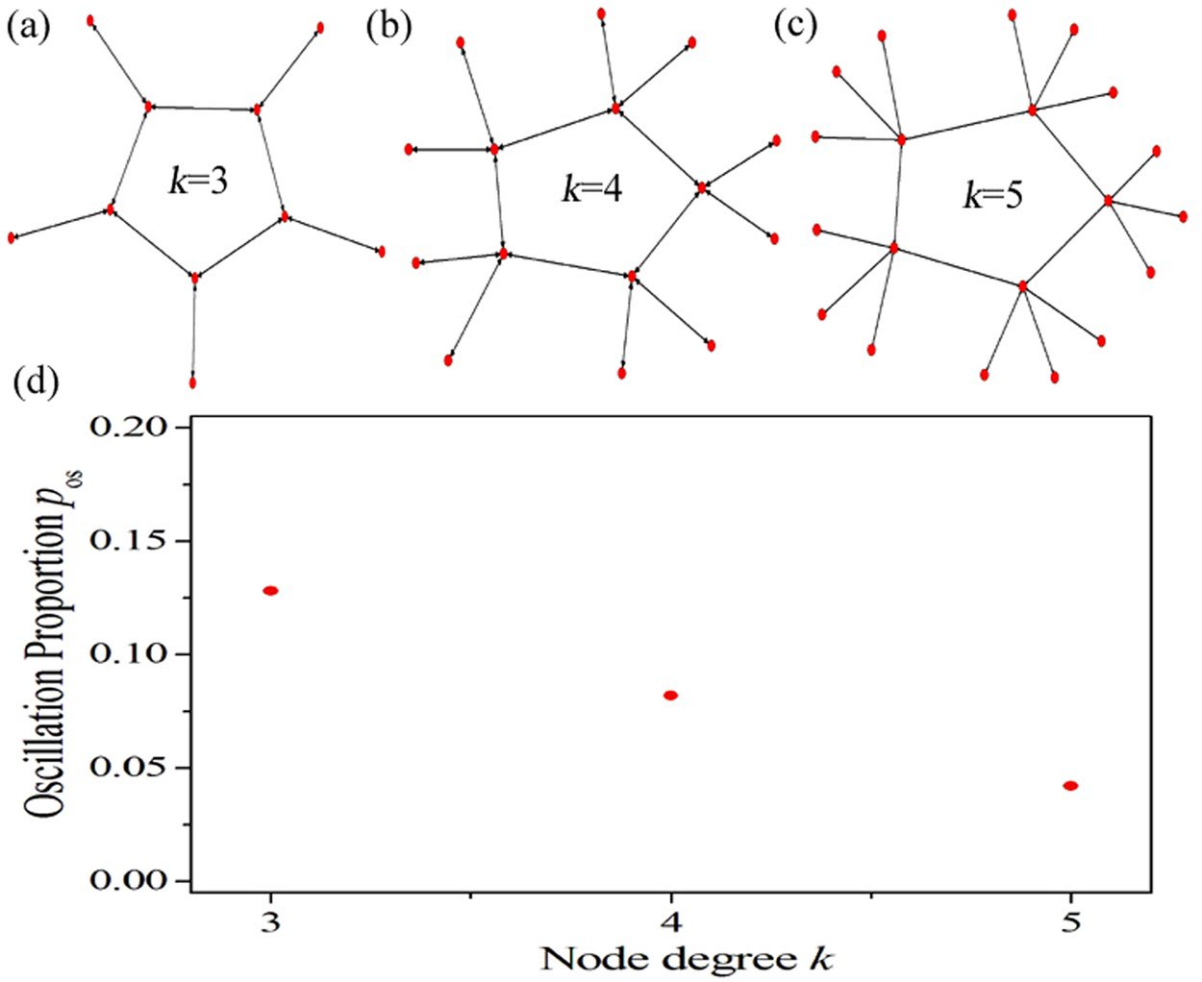
(**a,b,c**) The artificial 1D periodic excitable rings with different node degrees $k$. (**a**) $k\,=\,3$. (**b**) $k\,=\,4$. (**c**) $k\,=\,5$. The lengthes of these 1D periodic excitable rings are all fixed at 5 (i.e., there are 5 nodes in each excitable ring). The outside linking nodes increase remarkably as node degree $k$ is increased. (**d**) The dependence of the oscillation proportion ${p}_{{\rm{os}}}$ on the node degree $k$ in the artificial 1D periodic excitable rings of panels (a), (b) and (c). System parameters are chosen as $a\,=\,0.90$, $b\,=\,0.04$, $\varepsilon \,=\,0.04$ and $D\,=\,0.30$. One hundred independent numerical simulations are performed for each node degree $k$, and the random initial condition is utilized.

In the above paragraphs, we have studied the influence of node degree on the spatiotemporal dynamics of PSO in EHRNs and reveal a possible mechanism, which is the change of APL of network induced by the node degree. However, another potential determinant, i.e. the invasion of 1D Winfree loop (or we can say the invasion of oscillation source) from the outside linking nodes, should also be considered. We use the DPAD pattern shown in Fig. 2(a) to give further explanation. In Fig. 2(a) a 1D Winfree loop composed by nodes $79\to 32\to 49\to 23\to 96\to 79$ is discovered as the oscillation source supporting the PSO. The outside nodes, such as 40, 47, 91, 62, 44, are linking to the nodes in the loop. More importantly, these outside linking nodes do will impact on the formation of 1D Winfree loop. To confirm this point of view, the artificial 1D periodic excitable rings with different node degrees k are constructed, which are shown in Fig. 4(a)–(c), respectively (as shown in Fig. 4(a) for k = 3, shown in Fig. 4(b) for k = 4 and shown in Fig. 4(c) for k = 5). The lengthes of these 1D periodic excitable rings are all fixed at 5 (i.e., there are 5 nodes in each excitable ring). The outside linking nodes increase remarkably as node degree *k* is increased. With suitable initial conditions, excitable waves can propagate unidirectionally along the periodic excitable rings to form 1D Winfree loops. Due to the existence of the oscillation sources, PSOs can emerge in these rings. Figure 4(d) displays the dependence of the oscillation proportion *p*_os_ on the node degree *k* in these artificial 1D periodic excitable rings. System parameters are chosen as *a* = 0.90, *b* = 0.04, *ε* = 0.04 and *D* = 30. One hundred independent numerical simulations are performed for each node degree *k*, and the random initial condition is utilized. It is shown that the oscillation proportion *p*_os_ decreases gradually as the node degree *k* is increased. This means that these increased outside linking nodes do will promote the chance to invade the excitable ring to hinder the formation of 1D Winfree loop. Without the supporting from the oscillation source, fewer PSOs or even no PSO can be observed. This is the another potential mechanism, why the oscillation proportion *p*_os_ in EHRNs decreases as the node degree *k* is increased. Consequently, the results shown in Fig. 3(d) can be observed. Based on these discussions we can declare that the node degree, which is another major manner in regulating the network structure, can significantly influence the spatiotemporal dynamics of PSO in EHRNs. Two possible determinants behind the influence induced by the node degree are revealed. The one is the change of APL of network, and the other is the invasion of 1D Winfree loop from the outside linking nodes.

According to the investigation in this section we can conclude that the system size and the node degree are two major manners in regulating the network structure, and can impact the spatiotemporal dynamics of PSO in EHRNs remarkably. Importantly, two possible mechanisms are revealed. The PSO influenced by the network structures are induced not only by the change of APL of network (caused by the the system size or the node degree), but also by the invasion of 1D Winfree loop from the outside linking nodes (only caused by the node degree).

### The Influence Of System Parameters On PSO In EHRNs And The Related Key Determinants

**Figure 5:**
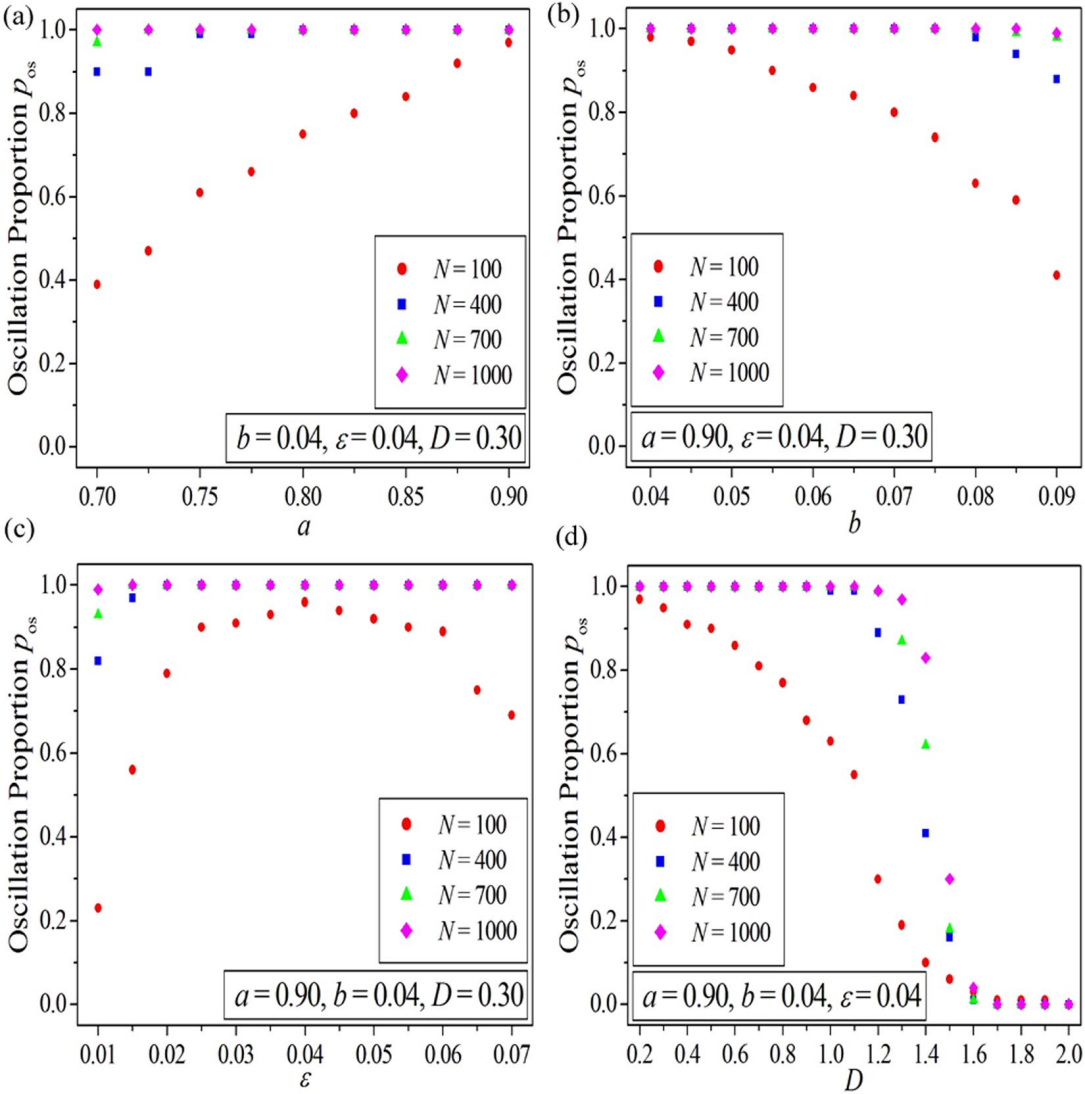
The dependence of the oscillation proportion ${p}_{{\rm{os}}}$ on the system parameters $a$ (**a**), $b$ (**b**), $\varepsilon$ (**c**) and $D$ (**d**) for different system sizes $N$ in EHRNs (shown by red dots for $N\,=\,100$, shown by blue squares for $N\,=\,400$, shown by green triangles for $N\,=\,700$ and shown by pink diamonds $N\,=\,1000$). The node degree is fixed at $k\,=\,3$. Other parameters are fixed and are marked in the corresponding panels.

In this section, we investigate the influence of system parameters on the spatiotemporal dynamics of PSO in EHRNs. Here we still use the oscillation proportion *p*_os_ as the indicator. Figure 5(a)–(d) display the dependence of the oscillation proportion *p*_os_ on the system parameters *a*, *b*, *ε* and *D* respectively for different system sizes *N* in EHRNs (as shown by red dots for *N* = 100, shown by blue squares for *N* = 400, shown by green triangles for *N* = 700 and shown by pink diamonds *N* = 1000). The node degree is fixed at *k* = 3. Other parameters are fixed and are marked in the corresponding panels. We first discuss the results obtained for small system size (i.e., shown by red dots in Fig. 5(a)–(d) for *N* = 100) to reveal the influence of system parameters on PSO in EHRNs and the related key determinants. Figure 5(a) exhibits the relationship between the oscillation proportion *p*_os_ and the parameter *a*. It is shown that the oscillation proportion *p*_os_ increases monotonically as the parameter *a* is increased. Contrary to the results obtained for the parameter *a*, the *p*_os_∼*b* relationship is plotted and is displayed in Fig. 5(b), in which the oscillation proportion *p*_os_ decreases monotonically as the parameter *b* is increased. Figure 5(c) reveals the dependence of the oscillation proportion *p*_os_ on the relaxation parameter $\varepsilon$. It is shown that, as the relaxation parameter $\varepsilon$ is increased from 0.01 to 0.07 gradually, the oscillation proportion *p*_os_ initially increases, then passes through a maximum, and finally decreases. The relationship between the oscillation proportion pos and the coupling strength D is displayed in Fig. 5(d), where the oscillation proportion *p*_os_ decreases gradually from 0.97 (corresponding to $D\,=\,0.20$) to 0.0 (corresponding to $D\,=\,2.0$) as the coupling strength *D* is increased.

Now we try to explain the dependence of the oscillation proportion *p*_os_ on the system parameters *a*, *b*, *ε* and *D* for small system size (i.e., the results shown by red dots in Fig. 5(a)–(d) for $N\,=\,100$). As we have stated above the formation of 1D Winfree loop, which exists as the oscillation source, is the key mechanism for maintaining the PSO in EHRNs. Moreover, as we know, nodes in excitable complex network must be excited in sequence. Consequently, the excitable wave must propagate forward along the shortest path in the network. The 1D Winfree loop, which can self-organize as the oscillation source to support the PSO in excitable complex network, should also obey this shortest path rule. This implies that the length of 1D Winfree loop should be as short as possible (i.e., the number of nodes in 1D Winfree loop should be as small as possible). However, as the existence of the refractory period of excitable dynamics, which is determined by the system parameters, the 1D Winfree loop cannot self-organize on a too small size topological loop. This means that there must be a minimum 1D Winfree loop at a given set of system parameters. Based on these discussions we can speculate that the minimum 1D Winfree loop will play a key role in interpreting the influence of system parameters on the spatiotemporal dynamics of PSO in EHRNs. Consequently, the dependence of the minimum 1D Winfree loop length on the system parameters is the crucially point, and needs to be exposed.

**Figure 6:**
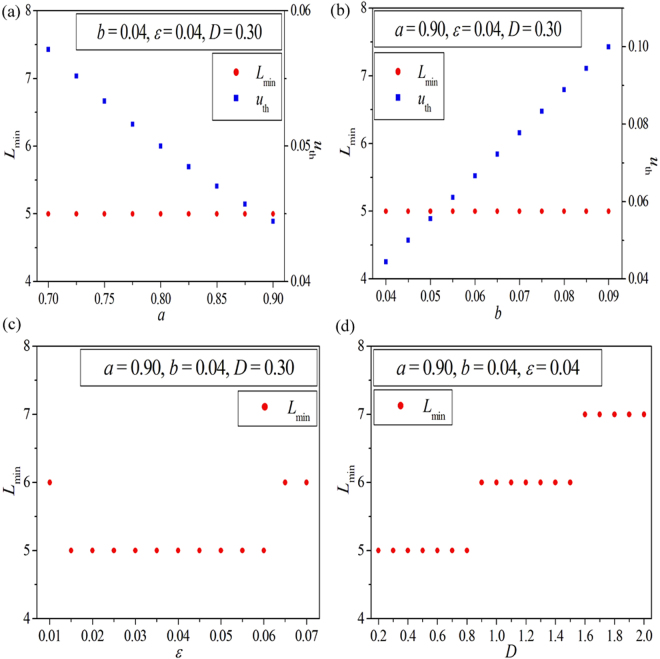
The relationship between the minimum 1D Winfree loop length ${L}_{{\rm{\min }}}$ and the system parameters $a$ (shown by red dots in (**a**)), $b$ (shown by red dots in (**b**)), $\varepsilon$ ((**c**)) and $D$ ((**d**)). The node degree of 1D Winfree loop is fixed at $k\,=\,3$. Other parameters are fixed and are marked in the corresponding panels. In panels (a) and (b), the left axis and the right axis denote the minimum 1D Winfree loop length ${L}_{{\rm{\min }}}$ and the excitation threshold of the Bär-Eiswirth model ${u}_{{\rm{th}}}$, respectively, and the blue squares represent the dependence of the excitation threshold ${u}_{{\rm{th}}}$ on the system parameters $a$ and $b$.

Figure 6(a)–(d) display the dependence of the minimum 1D Winfree loop length *L*_min_ on the system parameters *a*, *b*, *ε* and *D*, respectively. The node degree of 1D Winfree loop is fixed at $k\,=\,3$. Other parameters are fixed and are marked in the corresponding panels. Here we should mention that the length of a given 1D Winfree loop *L* can be approximately calculated by the formula $L\approx T\,\ast \,V$, where *T* is the oscillation period of the local excitable node and $V$ is the propagating speed of the excitable wave along the 1D Winfree loop. Due to the existence of the refractory period of excitable dynamics, there is a minimum oscillation period ${T}_{{\rm{\min }}}$, which approximately equals to the refractory period ${T}_{f}$. Consequently, the length of minimum 1D Winfree loop can be estimated approximately by the formula ${L}_{{\rm{\min }}}\approx {T}_{{\rm{\min }}}\,\ast \,V\approx {T}_{f}\,\ast \,V$. Here, the refractory period of the local excitable node ${T}_{f}$ and the propagating speed of the excitable wave *V* are decided by the system parameters. Based on the above discussion, we can find that the minimum 1D Winfree loop length, which is related to the refractory period of the local excitable node ${T}_{f}$ and the propagating speed of the excitable wave *V*, is largely determined by the system parameters and is independent of the initial conditions. Whatever the initial condition is, the constant minimum 1D Winfree loop length should be obtained.

Based on the results revealed in Fig. 6, now we can explain the influence of system parameters on PSO in EHRNs for small system size (i.e., the results shown by red dots in Fig. 5(a)–(d) for *N* = 100). The red dots in Fig. 6(a) show the dependence of the minimum 1D Winfree loop length *L*_min_ on the parameter *a*. It is shown that the minimum 1D Winfree loop lengthes are all fixed at ${L}_{{\rm{\min }}}\,=\,5$ in the whole interval of parameter *a*, which indicates that *L*_min_ is independent of *a* in this parameter region. However, the oscillation proportion *p*_os_ increases as the parameter *a* is increased (as shown by red dots in Fig. 5(a) for the *p*_os_∼*a* relationship). This means that the mechanism of the PSO in EHRNs influenced by the parameter *a* is not the minimum 1D Winfree loop. However, another key factor, i.e., the excitation threshold, which is related to the parameter *a*, should also be considered. As mentioned above, the excitation threshold of Bär-Eiswirth model is determined by ${u}_{{\rm{th}}}=\frac{b}{a}$. By increasing the parameter *a*, the excitation threshold of the local excitable dynamics will decrease (as shown by the blue squares in Fig. 6(a)), which can effectively improve the excitability of local excitable nodes and the wave propagation in excitable complex networks. This is beneficial for the spatiotemporal dynamics of PSO and can promote the emergence of PSOs in excitable complex networks. Consequently, the oscillation proportion *p*_os_ in EHRNs will increase as the excitation threshold is decreased (induced by the increase of the parameter *a*). And the dependence of the oscillation proportion *p*_os_ on the parameter a for small system size can be observed (as shown by red dots in Fig. 5(a) for *N* = 100).

Figure 6(b) displays the dependence of the minimum 1D Winfree loop length *L*_min_ (shown by the red dots) and the excitation threshold ${u}_{{\rm{th}}}$ (shown by the blue squares) on the system parameter *b*. Similar to the results obtained for the parameter *a*, the *L*_min_ fixes at 5 in the whole interval of the parameter *b*, and the excitation threshold increases as the parameter *b* is increased. The increase of excitation threshold is harmful for the excitability of local excitable nodes and the wave propagation in the network, which will hinder the formation of PSO in excitable complex networks. As a result, the oscillation proportion *p*_os_ in EHRNs decreases remarkably as the excitation threshold is increased (induced by the increase of the parameter *b*). And the PSO in EHRNs influenced by the parameter *b* for small system size can be obtained (as shown by red dots in Fig. 5(b) for $N\,=\,100$). Based on the above discussions we can declare that the system parameters *a* and *b* can effectively influence the spatiotemporal dynamics of PSO in EHRNs. The immediate determinant is the excitation threshold, which is decided by these two system parameters. So we call it as the excitation threshold determined PSO in EHRNs. However, this kind of influence degenerates as the system size expands. The blue squares, the green triangles, and the pink diamonds in Fig. 5(a) and (b) exhibit the dependence of the oscillation proportion ${p}_{{\rm{os}}}$ on the system parameters $a$ and *b* obtained at three larger system size *N* = 400, *N* = 700, and *N* = 1000, respectively. It is shown that the excitation threshold determined PSO in EHRNs degenerates as the system size is expanded.

Now we discuss the key factor of PSO in EHRNs influenced by the relaxation parameter $\varepsilon$ and the coupling strength $D$. Figure 6(c) shows the dependence of the minimum 1D Winfree loop length *L*_min_ on the relaxation parameter $\varepsilon$. As $\varepsilon$ is increased from 0.01 to 0.07 gradually, *L*_min_ initially decreases from 6 to 5, then stays at this level, and finally increases to 6 again. As we know the larger the *L*_min_ is, the harder for 1D Winfree loop to self-organize in the network supporting the PSO. Consequently, the oscillation proportion *p*_os_ influenced by the relaxation parameter $\varepsilon$ should initially increases, then passes through a maximum, and finally decreases. And this opposite trend has been confirmed in Fig. 5(c) (as shown by red dots for N = 100). Similar result is obtained for the coupling strength *D*. Figure 6(d) exhibits the relationship between the minimum 1D Winfree loop length Lmin and the coupling strength *D*. It is shown that, as *D* is increased, Lmin increases gradually, which will cause the decrease of the oscillation proportion *p*_os_ influenced by the coupling strength *D* (see red dots in Fig. 5(d) for N = 100). This further confirms the opposite trend between the oscillation proportion *p*_os_ and the minimum 1D Winfree loop length Lmin. Based on the results revealed in Fig. 6(c)–(d) and Fig. 5(c)–(d), we can assert that the relaxation parameter $\varepsilon$ and the coupling strength $D$ can effectively influence the spatiotemporal dynamics of PSO in EHRNs. The immediate determinant is the length of minimum 1D Winfree loop, which is decided by these two parameters. Hence, we call it as the minimum 1D Winfree loop determined PSO in EHRNs. However, this kind of influence also degenerates as the system size expands. The blue squares, the green triangles, and the pink diamonds in Fig. 5(c) and (d) exhibit the dependence of the oscillation proportion pos on the parameters $\varepsilon$ and $D$ obtained at three larger system size *N* = 400, *N* = 700, and *N* = 1000, respectively. It is shown that the minimum 1D Winfree loop determined PSO in EHRNs degenerates as the system size is expanded.

Based on the investigation in this part, we have discovered that the system parameters have remarkable influence on the spatiotemporal dynamic of PSO in EHRNs. Specifically, two distinct determinants, i.e., the excitation threshold and the minimum 1D Winfree loop determined PSO in EHRNs, have been exposed explicitly, which are otherwise deeply hidden behind the system parameters.

## Conclusion

In this paper, we have systematically investigate the influence of network structures and system parameters on the spatiotemporal dynamics of PSO in EHRNs. Firstly, a PSO emerging in the EHRN is presented. By using the DPAD method, the 1D Winfree loop is revealed as the oscillation source supporting the PSO, and the accurate wave propagation pathways from the oscillation source to the whole network are exposed clearly. Then, the oscillation proportion *p*_os_ is introduced, and is used as the order parameter to quantitatively study the influence of network structures and system parameters on the PSO in EHRNs. Phenomenally, we have found that network structures and system parameters have significant impacts on PSO. Importantly, the corresponding mechanisms are revealed, which are otherwise deeply hidden behind the network structures and the system parameters. PSO influenced by the network structures are induced not only by the change of APL of network, but also by the invasion of 1D Winfree loop from the outside linking nodes. Moreover, PSO influenced by the system parameters are determined by the excitation threshold and the minimum 1D Winfree loop. Finally, we uncovered that the excitation threshold and the minimum 1D Winfree loop determined PSO will degenerate as the system size is expanded.

Self-sustained oscillations in excitable complex networks are very important issues in wide practical fields, especially in neuronal networks and brain systems. A systematical investigation of the influence of network structures and system parameters on the spatiotemporal dynamics of periodically self-sustained oscillation in excitable homogeneous random networks and the related mechanisms are expected to be useful both for theoretical understandings and practical applications. Specifically, we have revealed four key factors in determining the emergence of periodically self-sustained oscillations in excitable homogeneous random networks, i.e., the system size, the node degree, the excitation threshold, and the minimum 1D Winfree loop length. According to these four key determinants, people can probably predict the emergence of oscillations in excitable complex networks, neuronal networks and brain systems. Furthermore, by using these four key determinants, people can effectively regulate the emergence of oscillations. If oscillations are beneficial and are needed in these systems, people can promote the emergence of oscillations by increasing the system size, decreasing the node degree, decreasing the excitation threshold, or selecting the system parameters with smaller minimum 1D Winfree loop length. If oscillations are harmful and needs to be inhibited, opposite operations can be performed. We do hope our work will be a useful supplement to the previous contributions and will have a helpful impact in related fields.
